# African-American inflammatory bowel disease in a Southern U.S. health center

**DOI:** 10.1186/1471-230X-10-104

**Published:** 2010-09-09

**Authors:** Hemanth Veluswamy, Kunal Suryawala, Ankur Sheth, Shannon Wells, Erik Salvatierra, Walter Cromer, Ganta V Chaitanya, Annette Painter, Mihir Patel, Kenneth Manas, Ellenmarie Zwank, Moheb Boktor, Kondal Baig, Balaji Datti, Michael J Mathis, Alireza Minagar, Paul A Jordan, Jonathan S Alexander

**Affiliations:** 1 Dept. of Molecular & Cellular Physiology, 1501 Kings Highway, Shreveport, LA, 71130-3932, USA; 2 LSUHSC-S Gastroenterology & Hepatology, 1501 Kings Highway, Shreveport, LA, 71130-3932, USA; 3 LSUHSC-S Dept. of Molecular & Cellular Physiology, 1501 Kings Highway, Shreveport, LA, 71130-3932, USA; 4 LSUHSC-S Cell Biology & Anatomy, 1501 Kings Highway, Shreveport, LA, 71130-3932, USA; 5 LSUHSC-S Information Services, 1501 Kings Highway, Shreveport, LA, 71130-3932, USA; 6 LSUHSC-S Neurology, 1501 Kings Highway, Shreveport, LA, 71130-3932, USA

## Abstract

**Background:**

Inflammatory Bowel Diseases (IBD) remain significant health problems in the US and worldwide. IBD is most often associated with eastern European ancestry, and is less frequently reported in other populations of African origin e.g. African Americans ('AAs'). Whether AAs represent an important population with IBD in the US remains unclear since few studies have investigated IBD in communities with a majority representation of AA patients. The Louisiana State University Health Sciences Center in Shreveport (LSUHSC-S) is a tertiary care medical center, with a patient base composed of 58% AA and 39% Caucasian (W), ideal for evaluating racial (AA vs. W) as well and gender (M vs. F) influences on IBD.

**Methods:**

In this retrospective study, we evaluated 951 visits to LSUHSC-S for IBD (between 2000 to 2008) using non-identified patient information based on ICD-9 medical record coding (Crohn's disease 'CD'-555.0- 555.9 and ulcerative colitis 'UC'-556.0-556.9).

**Results:**

Overall, there were more cases of CD seen than UC. UC and CD affected similar ratios of AA and Caucasian males (M) and females (F) with a rank order of WF > WM > AAF > AAM. Interestingly, in CD, we found that annual visits per person was the highest in AA M (10.7 ± 1.7); significantly higher (* -p < 0.05) than in WM (6.3 ± 1.0). Further, in CD, the female to male (F: M) ratio in AA was significantly higher (*- p < 0.05) (1.9 ± 0.2) than in Caucasians (F:M = 1.3 ± 0.1) suggesting a female dominance in AACD; no differences were seen in UC F: M ratios.

**Conclusion:**

Although Caucasians still represent the greatest fraction of IBD (~64%), AAs with IBD made up >1/3 (36.4%) of annual IBD cases from 2000-2008 at LSUHSC-S. Further studies on genetic and environments risks for IBD risk in AAs are needed to understand differences in presentation and progression in AAs and other 'non-traditional' populations.

## Background

Crohn's disease (CD) and ulcerative colitis (UC), the major forms of inflammatory bowel disease (IBD), are characterized clinically by diarrhea, weight loss and fever as well as endoscopic, radiologic, histopathologic findings and biochemical markers (e.g. perinuclear anti-neutrophil cytoplasm (p-ANCA), anti-*Saccharomyces cerevisiae *(ASCA) and IBD-specific p-ANCA markers) [1]. The development of IBD is thought to depend on several factors including genetic background, environmental influences e.g. dietary "hygiene", parasite burden and subclinical infectious diseases [2-5]. These factors lead to a complex overall distribution of IBD, with some described patterns in disease prevalence. IBD is considered a disease of developed nations especially Northern Europe and the United States, usually of colder climates with increased incidence as distance from the equator increases [6-10]. IBD is now increasingly reported in non-classical populations and in developing regions such as Asia, the Mid-East and in Africa [11,12].

In a study of central-African (Ghana) black patients [13] CD was viewed as a common, but often under-diagnosed condition, while Zaahl et al. [14] have reported that UC is relatively uncommon in South African blacks. In the US, IBD, (especially CD) is still regarded as a syndrome affecting individuals of European descent, but there is increasing evidence for IBD in the Hispanic and African American (AA) populations [15].

It is possible that clinical features of IBD may differ in AAs, leading to misdiagnosis and under-reporting of 'minority' IBD [15]. Eidelwein et al. [16] reported that pediatric CD was possibly more severe in AAs, often presenting with lower body mass scores, blood sedimentation rates and hemoglobin (Hb). AAs may also require more treatment (e.g. steroids, anti-TNF-α) to manage their IBD. In a study of pediatric/adolescent AA cases, White et al. [17], found that AAs were older at the time of diagnosis/onset, had a greater incidence of CD (>UC>indeterminate colitis), and lower Hb. Conversely, a meta-analysis of 8 studies with >2000 individuals suggested that no race-based differences exist in IBD susceptibility [18]. However, regional studies may suggest differences in IBD in AA populations exist. Ogunbi et al. report a CD incidence in AAs of 7-12 per 100,000 and 5-7 per 100,000 for UC [19]. In African populations, Shapira and Tamir [20] suggested that African CD incidence equals or exceeds that in European-Americans, consistent with underestimates in both African or AA IBD.

Incongruities about the incidence of IBD in AA populations may thus reflect low sampling, geographical influences, variations in disease presentation, environmental and socioeconomic factors that confound diagnosis and ultimately treatment. Gender-based differences in IBD have also been reported [21]; the F: M ratio in IBD may also be affected by urban/rural status [22-24] and may be changing in recent years [25].

### Sample Population of Shreveport/LSUHSC-S

One important limitation in this type of study is adequate sampling, in this case, ideally selecting populations in which AA patients at least match, or even exceed that of Caucasians. Our study carried out at the LSU Health Sciences Center in Shreveport (in accordance with HIPAA regulations regarding privacy) should approximate local ethnic profiles in Shreveport,/Arkansas-Louisiana-Texas region ('*'Arklatex''*). The racial composition of Shreveport has been >50% AA since 2002 [26]; the self-identified ethnic background of patients at LSUHSC-S (1999-2008) was 58.11% AA, and 39.42% Caucasian, (plus 1.43% Hispanic and 1.04% Asian, Native American plus other groups). Our goal was to evaluate the proportion of IBD cases as related to race and gender at LSUHSC-Shreveport, LA, a hospital serving the Northwest LA region with a roughly equal racial (AA : W) ratio.

## Methods

This study was reviewed and approved by the LSUHSC-Shreveport Human Research Protection Program/Institutional Review Board (IRB). We used non-identified ICD-9 codes (international statistical classification of diseases) from information medical record codes for CD (555.0 - 555.9) and UC (556.0 - 556.9). For every year in the study, individual non-identified patients were evaluated for self-identified race, gender and ICD-9 coding, (each patient was counted only once annually). Ages of individual patients at the time of annual visits were also recorded. Cumulative cases of CD or UC visits per each group were also counted (independent of the number of patients). We then determined: 1) the annual number of patients for each code, 2) the number of hospital visits per group, 3) the age at visit for individual racial and 4) gender populations at the LSUHSC in Shreveport, LA, (between 2000 and 2008). Hospital visits per individual were calculated as the total number of hospital visits (separated by race and gender) for each specific condition (CD or UC) and dividing this number by the number of cases for each condition. One-way ANOVA analysis was used to determine statistical significance with Bonferroni Multiple Comparisons post-testing. Statistical significance between pairs of data was determined using unpaired student's t-test. Nonlinear regression was used to calculate correlation and determine fit for trend lines in graphs. Because Hispanic, Asian, and Native American populations constitute <1% of total case reports for ICD-9 codes, these groups were omitted from analysis; we compared Caucasian and African-American populations with a further sub-grouping for gender. Co-morbid conditions with CD and UC, including diabetes (ICD-9 code 250.0), multiple sclerosis (ICD-9 code 340), and arthritis, were also investigated.

## Results

**Figure 1:**
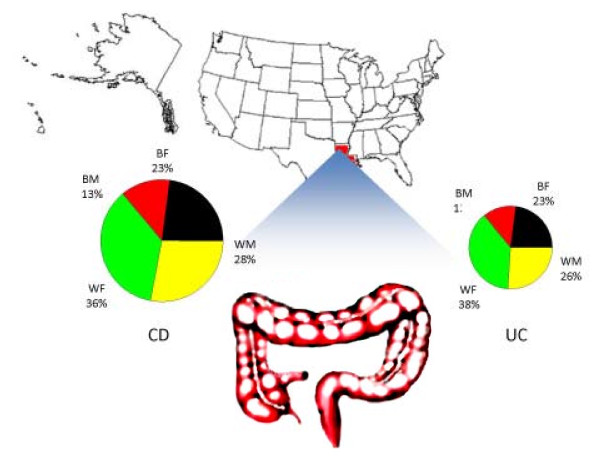
**Percent distribution of CD and UC in Shreveport, LA (2000-8)**. There is a strikingly similar distribution in CD and UC gender and race over a total of 9 years in Shreveport, LA. 665 CD patients and 286 UC patients were included in this study. There was variability and no correlation when data analyzed annually.

Data were collected on patients with CD and UC at LSUHSC-S between 2000-8. The compiled data set included: 1) the total number of annual visits, 2) the annual number of cases, 3) the age at visit for each disease and 4) these factors separated both by genders and by race at LSUHSC-S. Over the past 9 years, 665 patients with CD and 286 with UC treated at LSUHSC-S were included in this study. Of CD patients, 428 (64.36%) were Caucasian, and 237 (35.64%) were African-American. When the CD population was subdivided by race and gender, 240 (36.09%) were Caucasian Fs, 188 (28.27%) were Caucasian M, 150 (22.56%) were AA F, and 87 (13.08%) were AA M. Caucasians represented 63.99% (183) of UC patients, while African-Americans comprised 36.01% (103) in this study. When UC patients were subdivided by race and gender, 38.46% (110) were Caucasian F, 25.52% (73) were Caucasian M, 23.08% (66) were AA F, and 12.94% (37) were AA M (Fig. 1). It is important to point out that because our study only examined unidentified hospital records, the results only reflect institutional trends; and calculation of true regional or geographical incidences would require additional data on patient residence.

### Average age at hospital visit

The average age (at hospital visit) for both CD and UC was also studied. These data only reflect the average patient age at hospital visit, and do not infer the age of initial diagnosis. (CD: AA male 30.1 ± 1.73, A female 35 ± 1.42, WM 31.28 ± 1.19, WF 34.67 ± 1.14); (UC: AAM 38.27 ± 3.7, AAF 39.7 ± 2.12, WM 38.7 ± 2, WF 41.4 ± 1.6 AVG., ± S.E.). There was no statistical significance in average age amongst the population groups studied within each respective disease. When comparing CD to UC, at LSUHSC-Shreveport we did find that the average age at time of visit for CD to be significantly lower than for UC patients amongst Caucasian females (*, p < 0.05) and Caucasian M (*, p < 0.05).

### Number of cases per race/gender group

**Figure 2:**
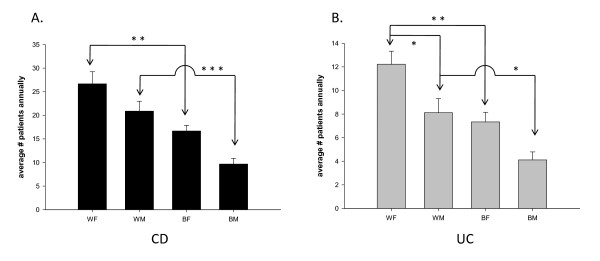
**Annual CD and UC cases by gender/racial group**. In fig. 2A the average number of cases seen annually for Crohn's disease is greater amongst Caucasian females than AA females (**, p < 0.01), and is also greater amongst Caucasian M when compared to AA M (***, p < 0.001). In fig. 2B the average number of cases seen annually for UC is greater amongst Caucasian females than Caucasian M (*, p < 0.05), Caucasian females than AA females (**, p < 0.01), and Caucasian M than AA M (*, p < 0.05). * Significantly different with p < 0.05; ** significantly different with p < 0.01; *** significantly different with p < 0.001 using one-way ANOVA, Tukey-Kramer multiple comparison.

**Figure 3:**
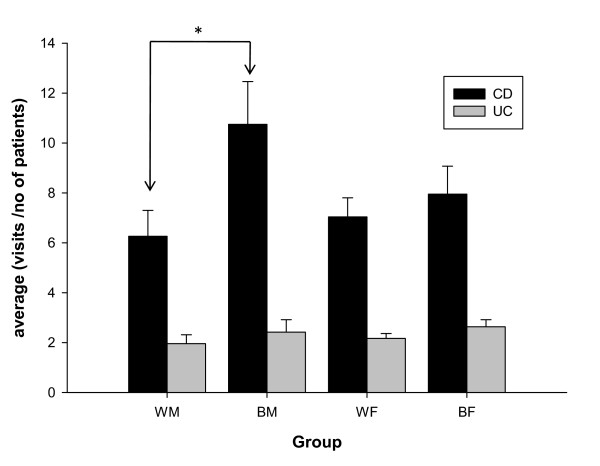
**Annual Crohn's Disease and Ulcerative Colitis visits per person**. AA males with Crohn's Disease made more annual visits to LSUHSC - Shreveport for treatment than Caucasian males with the disease. * Significantly different with p < 0.05 using one-way ANOVA, Bonferroni post-testing.

The annual number of cases per race/gender group was also compared for each condition. In both CD and UC, the average annual number of cases was highest amongst Caucasian F followed by Caucasian M followed by AA F, and finally the lowest number of cases was seen amongst AA M. When comparing this data in CD, Caucasian females had significantly more cases per year than AA females (**, p < 0.01). This was also seen when comparing Caucasian M to AAM (***, p < 0.001) (Fig. 2A). In UC, Caucasian F had significantly more cases per year than Caucasian M (*, p < 0.05) and AA F (**, p < 0.01). When comparing Caucasian M to AA M with UC, Caucasian M had more cases per year (*, p < 0.05) (Fig. 2B). Using the above-mentioned data, the average annual visits per person were calculated by taking the total number of visits per group yearly and dividing that number by the number of cases per respective group. Amongst Crohn's disease patients, AA M made more visits annually to LSUHSC-Shreveport than Caucasian M with this disease (*, p < 0.05) (Fig. 3). Further analysis for the trend of this disease was studied over 9 years for each group.

### Female: male ratios

**Figure 4:**
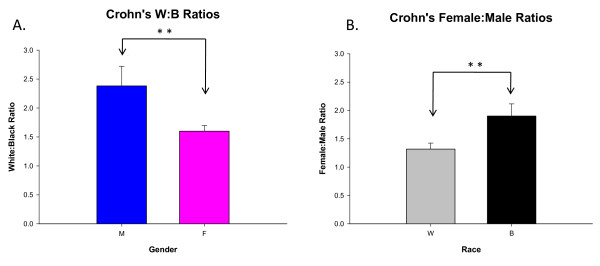
**Ratio comparison for Crohn's disease between A) Caucasians: AAs and B) females: males**. In fig. 4A, Crohn's affects more Caucasian than AA individuals (irrespective of genders). When comparing the W: B ratio, men were affected more than women (**, p < 0.01). In fig. 4B, Crohn's was seen to affect more women than men amongst both races. When comparing this ratio (F: M), AA individuals are affected more than Caucasian (**, p < 0.01). Results were compared using two-tailed unpaired student t-test.

**Figure 5:**
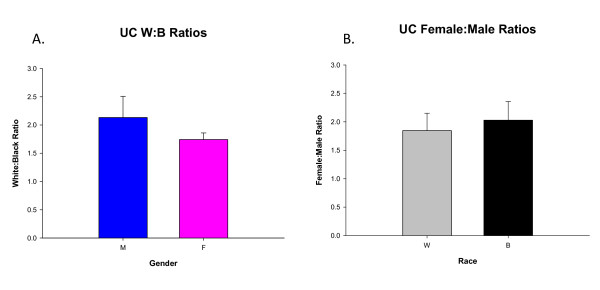
**Ratio comparisons for Ulcerative Colitis between A) Caucasians: AAs and B) females: males**. In fig. 5A, UC affects more Caucasian than AA individuals (irrespective of gender). When comparing this ratio (W: B) between men and women, no significance is seen. In fig. 5B, UC affects more women than men (irrespective of race). When comparing this ratio (F: M) between Caucasian and AA individuals, no significant difference is seen. Results were compared using two-tailed unpaired student t-test.

At LSUHSC-Shreveport, IBD affected more Caucasian (64%) than AA individuals (36%). We also compared the ratios of gender and race for both CD and UC. When comparing this ratio (W: B), men were affected more than women in CD (**, p < 0.01) (Fig. 4A). In UC when comparing the same ratio (W: B) between men and women, no significant difference was found (Fig. 5A). In both CD and UC women were more affected than men among both races. When comparing the F: M ratio, AAs were more affected than Caucasian (**, p < 0.01) amongst CD patients (Fig. 4B), however comparing the F: M ratio in UC, showed no significant difference (Fig. 5B).

## Discussion

IBD (Ulcerative Colitis, Crohn's disease) has long been considered as a group of diseases that affect primarily Caucasian populations [18,27], an impression which has recently been challenged by several studies. This concept remains uncertain for several possible reasons including under-sampling, racial or gender differences in IBD presentation, geographical or socio-economic differences influencing IBD etiology. For example, lower access to health care in AA could lead to under-reporting of IBD. Hospitalization for IBD is lower in AAs than in Caucasians [28], issues related to health coverage and reporting. Under-sampling in meta-analyses may also minimize or underestimate important trends, leading to erroneous conclusions regarding minority IBD incidence [29-31]. Minority under-representation at LSUHSC-Shreveport is unlikely. LSUHSC-Shreveport, part of the "charity" system is a university teaching hospital, providing equal care to patients regardless of ability to pay. We assume therefore that patient visits are not restricted by economic considerations, and all patients had equal access to health care. The ethnic/racial background at LSUHSC-S from 1999-2008 was 58.11% AA, and 39.42% Caucasian, (with 1.43% Hispanic and 1.04% Asian, Native American plus other groups). As of 2007, the Shreveport population was 43% Caucasian and 53.6% African-American (US average = 74.1% Caucasian, 12.4% = AA) [U.S. Census Bureau, 2005-2007 American Community Survey]. Between 2000-8, Caucasians made up 64% of IBD cases, ~ 36% of IBD cases were found in AAs [24].

Eidelwein et al. [16] also suggest differences may exist for the presentation and management of CD in AA compared to Caucasian pediatric patients (high sedimentation rate, low body mass z-score and [Hb], evidence of stricturing and a greater need for steroids and Infliximab to control disease). If IBD does emerge earlier in AA vs. Caucasians [32], it may be more aggressive [33]. With respect to other populations, Hispanics may have higher rates of UC (including co-morbid evidence of arthritis/uveitis) [34]. In UC, Hirschsprungs' disease (CPC 751.3) and schizophrenia (CPC 295) have been considered as co-morbid conditions. In CD, osteoporosis (CPC 733.0-.2) and amyloidosis (CPC 277) have also been reported [35]. IBD co-morbid conditions suggest that IBD may be systemic and polygenic, affecting numerous extra-intestinal targets [35], and that deviations from 'classical' (Caucasian)' IBD presentation might reflect race-based gene polymorphisms or environmental cues. All of these conditions as well as diabetes (CPC 250.0), multiple sclerosis (CPC340), and arthritis (CPC 714,715) were all investigated, but yielded too few co-morbid cases (<1%) for analysis based on patient records. Further studies are still required.

Several genes linked with development of IBD, have been compared in AA and Caucasian populations. In IBD, polymorphisms in Caspase recruitment domain-15 'CARD15' may dysregulate intestinal epithelial apoptosis and barrier [36]. Kugathasan et al. [37] correlated gene frequencies with racial background in IBD, finding fewer AA CARD15 mutations. Several CARD15 polymorphisms exist: CARD15 (2936insC) may mediate responses to LPS in AA CD [38]. Additional 'non-classical' CARD15 mutations e.g. A661P may drive AA CD, while the A725G CARD15 polymorphism may influence AA UC [39].

Another risk factor for CD is the NOD2 gene (Nucleotide-binding oligomerization domain containing 2). 3 polymorphisms in the NOD2/CARD15 gene (R702W, G908R and Leu1007finsC) (16q12) are risk factors for CD [40] by altering NF-kB influence over intestinal immune responses [30]. NOD2 polymorphisms are associated with ileal CD in Caucasians; in AA NOD2 may promote small bowel involvement, earlier onset, and fibro-stenotic disease uveitis and sacroiliitis [30].

Stenson and Snapper [41] cite several factors confounding accurate diagnosis of UC vs. CD including lack of standard criteria for diagnosis, inconsistent case histories, misdiagnosis, and misclassification. It is uncertain whether and how IBD disease activity is more severe in AAs. Many different approaches have been used to quantify IBD activity, but there is still no universally accepted scale of IBD severity. The Crohn's disease activity index (CDAI) provides a means of evaluating CD by assigning scores to subjective IBD measures (stool #, bleeding, and pain). Other studies use fistulae, extra-intestinal manifestations, surgical requirements and frequency of hospitalization as 'surrogate' markers of IBD. A scale for UC severity, using the extent of proximal colon involvement and clinical are also described [42-44].

It is noteworthy that the annual number of visits to LSUHSC for IBD was highest in AA males, (higher than Caucasian males) and may represent a significant 'surrogate' marker of disease, (similar to frequency of hospitalization). In our study between 2000-2008, the Caucasian: African-American patient ratio was 0.67:1. At the same time we found that more than 1/3 of IBD cases (35.64%) were seen in African-Americans, vs. 64.36% of IBD cases who were Caucasian (W). The Caucasian: African American IBD ratio is 1.8:1. Based on the ratios from our center, Caucasians were 2.68 times more likely to develop a form of IBD. We found a greater proportion of female cases in both CD and in UC with a male: female ratio of (0.7 ± 0.05, avg., ± SEM) in CD and a male: female ratio of (0.63 ± 0.07, avg. ± s.e.m.) in UC. This ratio is reversed from that reported by Kugathasan et al. [45] who reported male : female ratios in CD of 1.6, and in UC of 1.2 (predominantly a male disease) in pediatric populations. Kappelman et al. in a prevalence study of 9 million Americans found CD to be more prevalent among females (216 cases/100,000) than males (183 cases/100,000); that study found no significant difference in UC in adult populations [46]. Our data on gender in IBD are consistent with findings in Kappelmans' study regarding CD, but ours differ from their data on UC. Reasons for a female dominance in UC and CD in this study are not clear, but could reflect geographic or socio-economic factors.

Among UC patients at LSUHSC, 286 patients were seen over 9 years. Of these, 63.99% (n = 183) were Caucasian; of these, 38.46% (n = 110) were female and 73 (25.52%) were male. African-Americans comprised 36.01% (n = 103) of UC patients; of these 23.08% (n = 66) were female and 12.94% (n = 37) were male (Fig. 1). This means there are 2.33 times more CD than UC patients seen at the LSUHSC-Shreveport. Kappelman et al. [46] found a greater prevalence of UC (238 cases/100,000) over CD (201 cases/100,000), we found fewer UC cases than CD.

We found striking similarities in the fractional distributions between different populations of CD and UC patients included in the entirety of our study. In Crohn's disease, WF were 36.09%, WM were 28.27%, BF were 22.56%, and BM were 13.08%. In ulcerative colitis, WF were 38.46%, WM were 25.52%, BF were 23.08%, and BM were 12.94%. (There were no remarkable year-to-year differences between these groups).

Although the number of hospital visits at the LSUHSC-Shreveport for CD and UC have been increasing annually in Shreveport since 2000, total visits have also increased, and the fraction of cases for each condition has not changed significantly. Our results among these groups show that AA M had more hospital visits (per individual) for CD than other groups; this was not found when analyzing UC visits (Fig. 3). AA females had the 2^nd ^highest rate of hospital visits (per individual) for CD, (not significantly different vs. WF). While preliminary, hospital visits may represent a potential 'surrogate' marker of disease activity requiring additional study.

## Conclusions

In summary, we provide racial, gender and age statistics of IBD at the LSUHSC in Shreveport over the past 9 years. Our institution serves more AAs than any other racial group and provides an important site for opportunity to study IBD in African Americans. While Caucasians represented the most cases of IBD, AAs represented 36% of IBD cases with the highest annual visits among AA males. Classical assumptions about the prevalence and course of IBD in AAs may underlie the failure to suspect, diagnose, and treat IBD in this population.

## Competing interests

The authors declare that they have no competing interests.

## Authors' contributions

HV, performed data compilation and analysis, graphic and manuscript preparation. KS performed data compilation and statistical analysis, graphics and manuscript preparation. AS, performed data compilation and analysis, and worked on introduction and discussion. ES performed statistical analysis, and manuscript introduction and discussion. SW performed final data compilation, bibliographic, graphics and worked on manuscript introduction and discussion. AP coordinated and collected de-identified data sets from hospital records. GVC helped develop the concept for the paper, supervised statistical analysis, assisted in writing the introduction and discussion. KM coordinated fellow participation, assisted in writing the introduction for the manuscript. KB performed data analysis and statistics and contributed to introduction. EZ contributed to introduction and conclusion sections. MB assisted with data analysis, graphics and introduction. WS assisted in statistical analysis, graphics and in writing the manuscript. JMM assisted with data analysis, and discussion. BD initiated data collection and analysis (with AP). PJ assisted in developing the overall concept, interpreted data, wrote introduction, discussion and conclusions. AM interpreted data and assisted in writing conclusions. JSA developed the overall concept, supervised collaborators and wrote introduction, discussion and conclusions. All authors have read and approved the final manuscript.

## Pre-publication history

The pre-publication history for this paper can be accessed here:

http://www.biomedcentral.com/1471-230X/10/104/prepub
